# The mode and tempo of hepatitis C virus evolution within and among hosts

**DOI:** 10.1186/1471-2148-11-131

**Published:** 2011-05-19

**Authors:** Rebecca R Gray, Joe Parker, Philippe Lemey, Marco Salemi, Aris Katzourakis, Oliver G Pybus

**Affiliations:** 1 Department of Pathology, Immunology and Laboratory Medicine, University of Florida, Gainesville, FL, USA; 2 Emerging Pathogens Institute, University of Florida, Gainesville, FL, USA; 3 Kitson Consulting, Bristol, BS8 3UL, UK; 4 Department of Microbiology and Immunology, Katholieke Universiteit Leuven, Leuven, Belgium; 5 Department of Zoology, Oxford University, South Parks Road, Oxford, OX1 3PS, UK

**Keywords:** hepatitis C, substitution rate, virus evolution, Bayesian phylogenetics, molecular clock, relaxed clock, adaptation

## Abstract

**Background:**

Hepatitis C virus (HCV) is a rapidly-evolving RNA virus that establishes chronic infections in humans. Despite the virus' public health importance and a wealth of sequence data, basic aspects of HCV molecular evolution remain poorly understood. Here we investigate three sets of whole HCV genomes in order to directly compare the evolution of whole HCV genomes at different biological levels: within- and among-hosts. We use a powerful Bayesian inference framework that incorporates both among-lineage rate heterogeneity and phylogenetic uncertainty into estimates of evolutionary parameters.

**Results:**

Most of the HCV genome evolves at ~0.001 substitutions/site/year, a rate typical of RNA viruses. The antigenically-important *E1/E2 *genome region evolves particularly quickly, with correspondingly high rates of positive selection, as inferred using two related measures. Crucially, in this region an exceptionally higher rate was observed for within-host evolution compared to among-host evolution. Conversely, higher rates of evolution were seen among-hosts for functionally relevant parts of the *NS5A *gene. There was also evidence for slightly higher evolutionary rate for HCV subtype 1a compared to subtype 1b.

**Conclusions:**

Using new statistical methods and comparable whole genome datasets we have quantified, for the first time, the variation in HCV evolutionary dynamics at different scales of organisation. This confirms that differences in molecular evolution between biological scales are not restricted to HIV and may represent a common feature of chronic RNA viral infection. We conclude that the elevated rate observed in the *E1/E2 *region during within-host evolution more likely results from the reversion of host-specific adaptations (resulting in slower long-term among-host evolution) than from the preferential transmission of slowly-evolving lineages.

## Background

Rapidly-evolving RNA viruses that establish chronic infections, such as the human immunodeficiency virus (HIV) and the hepatitis C virus (HCV), appear to exhibit qualitatively different evolutionary dynamics when their genetic diversity is studied at different organisational scales [1,2]. Within-host evolutionary dynamics can be observed by comparing sequences that represent different virions sampled from a single infected individual over several years, whereas among-host evolution is revealed by collating sequences that each represent a different infected host. The most significant distinction between these two levels is that the evolution of among-host sequences is shaped by numerous genetic bottlenecks arising from transmission events, whereas that of within-host sequences is not.

The hepatitis C virus (HCV) (family *Flaviviridae*, genus *Hepacivirus*) infects more than 180 million people worldwide and is a leading global cause of liver disease and liver cancer [3,4]. Understanding the evolution of HCV has considerable medical relevance. For example, viral diversity is known to play a key role in determining both the outcome of long-term chronic infection and the likelihood of success of anti-viral drug therapy [5-10]. However, despite the wealth of HCV sequence data and the relevance of HCV genetic diversity to public health, many aspects of HCV molecular evolution are poorly understood, particularly in comparison to HIV, despite HCV having a higher overall global prevalence that HIV. One of the most fundamental aspects of HCV evolution is its genomic rate of molecular evolution. Previous estimates of HCV evolutionary rates have employed a number of different estimation methods, genome regions and scales of analysis, hindering direct comparisons, and few have systematically considered the variation in evolutionary rate along the whole HCV genome and its causes [11]. The HCV genome encodes a single polyprotein ~3000 amino acids in length, comprising three structural (*Core*, *E1 *and *E2*) and seven non-structural genes (*p7*, *NS2, NS3*, *NS4a, NS4b, NS5a *and *NS5b*) [12]. The *E2 *envelope glycoprotein contains 'hyper-variable regions' (HVRs) that are targeted by the human humoral immune response [13]. An additional limitation of previous studies is that they have typically assumed that evolutionary rates are constant among lineages and through time (known as the 'strict' or 'single rate' molecular clock hypothesis). Although statistical tests of this hypothesis for HCV are not always significant (e.g. [14,15]), such failures to reject the strict clock are most likely a reflection of small sample sizes, because larger HCV data sets indicate significant among-lineage rate variability [11,16]. Furthermore, previous studies have typically evaluated HCV rates using a single estimated phylogeny, thus ignoring an important source of statistical error (although [11] approximated this error through bootstrapping procedures). Many previous studies used non-phylogenetic methods (such as the relative-rates test) to estimate the HCV evolutionary rate, which are now known to be less efficient and potentially more biased than phylogenetic approaches [17].

Previous analyses of HCV molecular evolution also have been restricted to a single evolutionary scale. It has been demonstrated that the evolutionary dynamics of HIV differ substantially among levels of organisation [2,18]. For example, HIV-1 within-host evolutionary rates are higher [1] and more variable [19] than those among-hosts. Although the biological causes of these differences are as yet unknown, possible explanations include preferential transmission of slowly-evolving lineages, a decreasing within-host evolutionary rate through time, or viral reversion to variants of higher fitness upon transmission to a new host [1]. Crucially, it is not known whether these scale-dependent differences are peculiar to HIV-1 or whether other chronic viral infections, such those caused by HCV, exhibit similar behaviour. Detailed investigation of multi-level evolution in viral populations - for which much data are available - may help to build a more general understanding of this complex evolutionary phenomenon.

Here, for the first time, we quantify and compare the within- and among-host molecular evolution of whole HCV genomes. We avoid the methodological limitations outlined above by analysing whole genome sequences and employing a 'relaxed molecular clock' approach that explicitly models and estimates the level of rate variation among lineages [20]. This approach is implemented in a Bayesian inference framework [21] that incorporates phylogenetic uncertainty into estimates of evolutionary parameters. In addition to employing powerful methods of analysis, we study the entire HCV genome using a partition approach, thereby revealing how scale-dependent evolution affects different viral genome regions in different ways.

## Methods

### Datasets

We compiled three datasets, one representing only evolution within infected hosts, the other two representing evolution at the 'epidemiological' or among-host level. To maximise compatibility and statistical power, we sought data sets comprising sequences from the same subtype sampled over at least 20 years, and which contain complete or near-complete viral genomes.

Our within-host data set is based on HCV subtype 1b genomes obtained from 15 women who were all infected by a contaminated blood product (anti-D immunoglobulin) that had been generated from a single HCV-infected blood donation ([22]; coloured red in all figures). This data set comprises full-length genomic HCV sequences from 15 patients, sampled at two time-points, plus an additional sequence sampled in 1977 from the HCV-infected blood donation (*n *= 31; sampling dates range from 1977 to 2000; [23]. Since the contaminated blood product contained little viral diversity [24], all recipients were infected with very similar viral sequences; hence this data set represents - perhaps uniquely - 15 independent within-host evolutionary histories, yet contains no secondary transmission events. Among-lineage rate variation in these sequences therefore reflects in equal part variation in evolutionary rate among infected hosts and among different lineages within a host. Accession numbers and isolate sampling dates of all sequences used in this study are listed in Additional file 1, Table S1.

We obtained two data sets representing evolution at the among-host level. We collected all available HCV genotype 1 whole genome sequences from the Broad Institute database http://www.broadinstitute.org/annotation/viral/HCV. These genomes were split into two data sets, comprising 334 subtype 1a and 149 subtype 1b genomes, respectively. In all figures the subtype 1a data set is coloured green and the subtype 1b data set coloured blue. Sequences were primarily collected in the USA (n = 377) as well as from Switzerland (n = 88) and Germany (n = 18). To aid computational efficiency, the subtype 1a and 1b datasets were reduced in size, resulting in two final alignments comprising 63 subtype 1a and 54 subtype 1b sequences. Retained sequences were chosen such that the original temporal range of the initial dataset (1989-2008) was maintained, by randomly excluding sequences from over-represented years. Both among-host data sets represent HCV evolution across several decades of epidemic transmission, hence each branch in their phylogenies will represent a number of transmission events.

### Model selection procedure

To select the best-fitting evolutionary model for Bayesian MCMC inference, we performed an initial series of model selection analyses using BEAST v1.5.4. MCMC output was inspected for convergence by visual inspection of the chain and by calculation of effective sample size statistics, as implemented in Tracer v1.5 http://tree.bio.ed.ac.uk. Where necessary, MCMC operators were optimised by trial and error to improve chain mixing. Various different substitution, coalescent and molecular clock models were compared by calculating Bayes Factors (BF), which is the difference in log marginal likelihoods between two model combinations [25,26]. We calculated approximate marginal likelihoods for each model via importance sampling using the harmonic mean of the sampled likelihoods (with the posterior as the importance distribution; see [27]). Evidence against the null model (i.e. the model with lower marginal likelihood) is indicated by 2ln(BF) >3 (positive evidence) and >10 (strong evidence).

In all datasets, nucleotide sites were assigned to two partitions: (i) 1^st ^& 2^nd ^codon positions and (ii) 3^rd ^codon positions. Our preliminary analyses indicated that a good fit to the data was obtained by ascribing a separate HKY nucleotide substitution model and a separate gamma among-site rate heterogeneity model to each of the two codon partitions (equivalent to the model described in [28]). This substitution model was sufficiently computationally-efficient to permit MCMC convergence (data not shown) and was therefore used throughout the remainder of the study. For the within-host data set, phylogenetic priors were used to represent known epidemiological information about the transmission chain: specifically, all sequences from the infected patients (i.e. all except the single sequence from the infected blood donation) were constrained to be a monophyletic group, and a prior normal distribution was imposed on the date of the common ancestor of this group (mean = 25 years before present and variance = 1 year).

Both strict and relaxed molecular clock models were tested for each of the three datasets using the Bayes Factor test as described above. The uncorrelated lognormal relaxed molecular clock model (UCLN) was used, which provides an estimate of the 'coefficient of variation' statistic, representing the scaled variance in evolutionary rate among lineages (see [20] for details). This statistic is usefully interpreted as indicating the degree to which molecular evolution is 'clock-like'. A posterior distribution for the coefficient of variation that does not include zero indicates that the relaxed clock model provides a better fit to the data than the strict clock. Having chosen the optimal molecular clock model for each data set, we tested three different coalescent models: (i) constant population size, (ii) exponential growth, and (iii) the flexible Bayesian skyline plot (BSP) model. The optimal coalescent model was also chosen using Bayes Factors. Note that in this study, the coalescent model is being used as a prior distribution for phylogenies, whose parameters are marginalised and ignored, and not as an explicit model of the population under study.

### Genomic partition model

After the selection of the most appropriate molecular clock and coalescent models, each data set was split into 21 non-overlapping partitions 432 nt in length, beginning at the start codon of the *Core *gene. In order to estimate separate molecular clock parameters for each partition whilst simultaneously minimising estimation variance, we implemented a genomic partition model in BEAST v1.5.4 [21]. This model estimated separate molecular clock parameters and nucleotide frequencies for each partition, whilst all partitions share the same underlying nucleotide substitution model (as above), coalescent model parameters, and phylogenetic tree, thus making the most statistically efficient use of the available sequence information. XML files for performing these analyses are available on request. Partitions were kept of equal length (and not adjusted to coincide with gene boundaries) so that intra-genic variation could be measured and so that estimation uncertainty could be directly compared among genome regions. We deliberately ignored the alternate open reading frame in the *Core *gene, as its molecular evolution is under no selective constraints (e.g. [29]). This ORF therefore has no effect on the evolution of the HCV main reading frame

### Partition specific dn/ds analyses

It was computationally-impractical to calculate *dn/ds *ratios for each partition whilst simultaneously incorporating phylogenetic uncertainty, hence we began by estimating a maximum likelihood (ML) tree for each of the three datasets using PhyML, under a HKY model of nucleotide substitution with gamma distributed site variation. The ratio of replacement-to-silent nucleotide substitution (*dn/ds*) and the transition/transversion ratio were calculated separately for each of the 21 non-overlapping partitions defined above using PAML [30], given the ML tree and nucleotide alignment for each dataset.

## Results

### Model selection procedure

Table 1 shows the estimated statistical and evolutionary parameters for each model combination we investigated. For all three data sets, the relaxed clock model provided a much better fit than the strict clock model (BF>20). Although the choice of coalescent model did not significantly affect model fit (BF<3 for most comparisons), the MCMC under the constant size model failed to converge (data not shown). This is perhaps unsurprising, as phylogenies estimated from all data sets are star-like in shape (i.e. long terminal branches) and therefore poorly characterised by a constant-size coalescent model.

**Table 1:** Model Selection Analysis Results

Data set	Molecular clock model	**Coalescent Model**^**a**^	Marginal Log Likelihood	**Genomic rate of evolution**^**b**^**(×10**^**-3**^**subs./site/year)**	Relaxed clock coefficient of variation	Relative rate of evolution (codon positions 1 & 2)	Relative rate of evolution (codon position 3)
**Among-host (1a)**	Strict	Constant	-90901.35	1.30 (1.16 - 1.43)	*Not applicable*	0.72 (0.70 - 0.74)	1.56 (1.51 - 1.60)
	Relaxed	Constant	-90632.03	1.44 (1.00 - 1.84)	0.25 (0.21 - 0.30)	0.72 (0.70 - 0.74)	1.56 (1.51 - 1.60)
	Relaxed	Expo	-90632.39	1.47 (1.02 - 1.87)	0.24 (0.20 - 0.29)	0.72 (0.70 - 0.74)	1.56 (1.51 - 1.60)
	Relaxed	BSP	-90632.49	1.48 (1.09 - 1.84)	0.23 (0.20 - 0.27)	0.72 (0.70 - 0.75)	1.55 (1.51 - 1.60)

**Among-host (1b)**	Strict	Constant	-88288.70	1.04 (0.87 - 1.22)	*Not applicable*	0.66 (0.64 - 0.68)	1.68 (1.63 - 1.72)
	Relaxed	Constant	-88127.66	1.25 (0.73 - 1.73)	0.22 (0.18 - 0.26)	0.66 (0.64 - 0.68)	1.68 (1.64 - 1.72)
	Relaxed	Expo	-88125.80	1.24 (0.75 - 1.74)	0.21 (0.17 - 0.25)	0.66 (0.64 - 0.68)	1.68 (1.64 - 1.72)
	Relaxed	BSP	-88127.55	1.18 (0.67 - 1.65)	0.21 (0.17 - 0.25)	0.66 (0.64 - 0.68)	1.68 (1.63 - 1.72)

**Within-host**	Strict	Constant	-31070.20	1.12 (1.02 - 1.23)	*Not applicable*	0.58 (0.54 - 0.61)	1.85 (1.78 - 1.92)
	Relaxed	Constant	-31013.86	1.11 (0.97 - 1.23)	0.27 (0.19 - 0.37)	0.58 (0.54 - 0.61)	1.85 (1.78 - 1.92)
	Relaxed	Expo	-31013.56	1.13 (1.01 - 1.27)	0.30 (0.19 - 0.42)	0.58 (0.54 - 0.61)	1.85 (1.78 - 1.92)
	Relaxed	BSP	-31012.14	1.11 (0.98 - 1.24)	0.27 (0.19 - 0.34)	0.58 (0.54 - 0.61)	1.85 (1.78 - 1.92)

^a ^Constant = constant population size, Expo = exponential growth, BSP = Bayesian skyline plot

^b ^A single average rate of evolution estimated across the whole genome (no partitions)

For all three data sets, the estimated genomic rate of evolution was consistent among all of the coalescent models investigated under the relaxed clock assumption (Table 1). For the among-host subtype 1a data set, the mean genome-wide rate was estimated to be 1.44 - 1.48 × 10^-3 ^substitutions/site/year (among different coalescent models), while the equivalent rates for the subtype 1b data set were slightly lower (1.18 - 1.25 × 10^-3 ^substitutions/site/year). The within-host genome-wide evolutionary rate was lower still than the two among-host datasets (1.11 - 1.13 × 10^-3 ^substitutions/site/year). For all analyses, the lower confidence limit of the coefficient of variation statistic (which measures 'un-clock-likeness') was above zero, indicating statistically-significant variability in evolutionary rate among lineages. As expected for a continuous protein-coding region, the relative evolutionary rate of 1^st ^and 2^nd ^codon positions was significantly lower than that of the 3^rd ^codon position (two-tailed T-test *p *< 0.01).

### Genomic partition model

**Figure 1:**
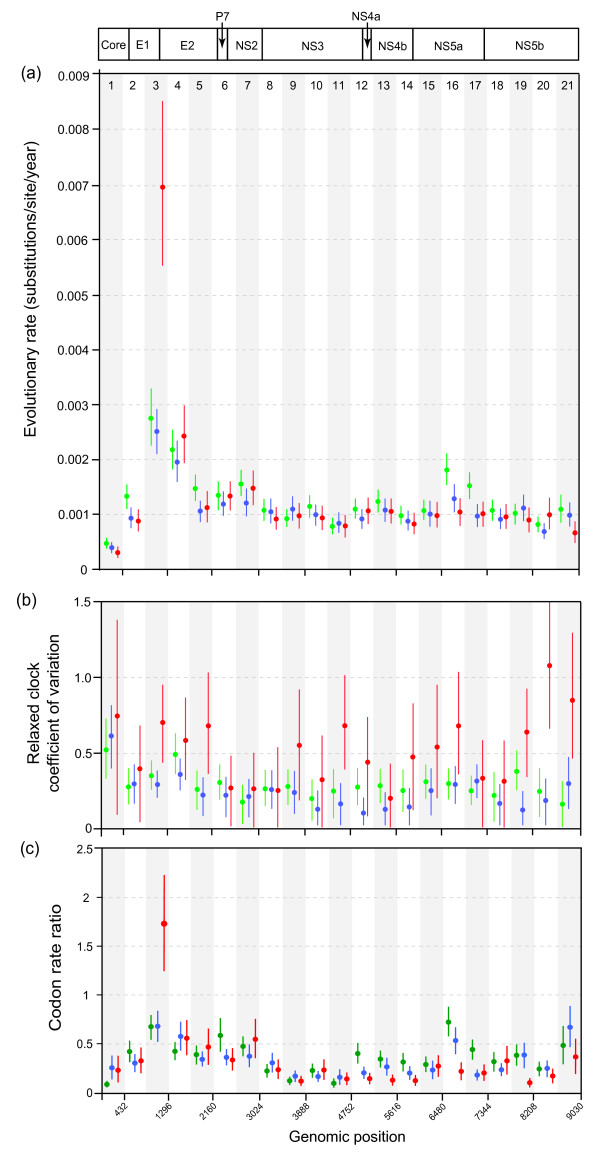
**The molecular clock behaviour of the HCV genome**. Three separate data sets are shown: among host subtype 1a (green), among host subtype 1b (blue) and within-host (red). Separate parameters were estimated for each of 21 partitions spanning the HCV coding region (see genome schematic and partition numbering at top of Figure). The alternating white and grey bars are for visual clarity only; nucleotide numbering according to the H77 reference genome is show at the bottom. (a) Estimated mean evolutionary rates. For each partition and data set, the vertical bar represents the range of the 95% HPD credible region and the circle represents the point estimate of the mean rate. (b) Estimated coefficient of variation (COV) parameters for each partition and data set, which represent the among-lineage rate heterogeneity. The vertical bars represent the range of the HPD credible region and the circle shows the estimated COV value. (c) Estimated codon rate ratio (CRR) values, which represent the ratio of the evolution rate at codon positions 1 and 2 to that at codon position 3. As before, the vertical bar represents the range of the 95% HPD credible region and the circle represents the point estimate of CRR.

In light of the model selection results, the relaxed molecular clock was used in the genomic partition analysis. Since neither the BSP nor the exponential coalescent model was statistically favoured, we chose the model that exhibited the best MCMC mixing behaviour (BSP for the among-host datasets; exponential for the within-host dataset). For each genomic partition, we estimated the following parameters: (i) mean substitution rate, (ii) relaxed clock coefficient of variation, (iii) the relative rate of codon positions 1+2 to that of codon position 3. The estimated values for each of these parameters are shown in Figure 1, with both the point estimate (dots) and the 95% highest posterior density (HPD) confidence limits (vertical lines) shown for each parameter in each partition.

Figure 1a shows clear trends in evolutionary rate variation across the genome. In the majority of partitions the mean evolutionary rate of the among-host subtype 1a data set was slightly higher than that of subtype 1b (in agreement with the whole genome values presented in Table 1). For most of the non-structural genes (*p7, NS2, NS3, NS4a, NS5a *and *NS5b*), mean evolutionary rate was consistently about 1.0 × 10^-3 ^substitutions/site/year, for all three datasets. However, notably higher rates were observed for the subtype 1a among-host data set, in the 16^th ^and 17^th ^partitions (3' end of the NS5a gene), which contain functionally important domains (see Discussion for details). Within the structural genes (*Core, E1 *and *E2*) estimated mean evolutionary rates were low for the *Core *region (0.28 - 0.43 × 10^-3 ^substitutions/site/year) and high in partitions 3 and 4 (the C-terminus of *E1 *and N-terminus of *E2*). Partition 3 corresponds to the location of the hyper-variable region HVRI (2.5 - 6.9 × 10^-3 ^substitutions/site/year). Partition 4, which includes the HVRII and III regions, also exhibited an elevated evolutionary rate in all three data sets (2.4 - 2.7 × 10^-3 ^substitutions/site/year). Overall, variation in mean evolutionary rate is greater among genome regions than among the three data sets, with one very notable exception: the evolutionary rate of partition 3 (containing the HVRI) is remarkably higher for the within-host data set than for the two among-host data sets. The G/C content at each codon position was similar among datasets, being higher at the 3^rd ^position than at the 1^st ^or 2^nd ^positions (Additional file 2, Figure S1), as previously reported [31].

For each partition, we also estimated the coefficient of variation (COV) statistic (Figure 1b). In general, the mean and variance of this parameter was significantly higher for the within-host dataset, hence among-branch rate variation is much higher in this data set than in the among-host data sets. In several partitions, the lower HPD confidence limit was close to zero, indicating that the strict clock hypothesis could not be excluded in these instances. Mean COV values for among-host data sets were typically around 0.2-0.3, in line with previous estimates for HIV [19] with the exception of the *Core *gene, which exhibited values >0.5. For the within-host dataset, COV estimates were also elevated for the last two genomic partitions (covering the C-terminus of *NS5b*).

We also estimated the ratio of the evolutionary rate at codon positions 1 & 2 to that at codon position 3 (the codon rate-ratio, CRR). This ratio can be computed concurrently with other molecular clock parameters and can be used to investigate selective pressures acting on gene sequences, because almost all changes at codon positions 1 & 2 are non-synonymous and the majority of changes at codon position 3 are synonymous. Estimates of the CRR for each genomic partition are shown in Figure 1c. In general, the CRR was low between partitions 8 and 20, corresponding to most of the non-structural genome region, indicating on average strong selective constraint. Partitions 16 and 21, however, have raised CRR values for the among-host data sets. CRR values are slightly higher in the *E1*, *E2 *and *NS2 *genes, with a particularly high ratio observed for the within-host data set in partition 3.

To determine whether the elevated evolutionary rate and correspondingly high CRR of partition 3 in the within-host data set was independent of the 3^rd ^codon position rate, we plotted the absolute rates for the 1^st^+2^nd ^versus 3^rd ^codon positions for each partition (Additional file 3, Figure S2). As expected, the 1^st^+2^nd ^position rate was much lower than the 3^rd ^position rate for all partitions in the among-host datasets. For partition 3 of intra-host dataset, the 1^st^+2^nd ^position rate (0.008 subs./site/year) was greater than the 3^rd ^position rate (0.005 subs./site/year). However, in this partition the 3^rd ^position rates for the intra-host datasets were also significantly elevated. The lowest 3^rd ^position rates were observed in partitions 1 and 21. This is consistent with the presence of RNA secondary structure (stem-loops) in these regions of the HCV coding region [32]. This structure will likely impose selective constraints on 3^rd ^codon positions, resulting in a lower evolutionary rate at silent sites. However, we observed no consistent effect of RNA secondary structure on CRR values: in partition 21 the CRR is raised, which could reflect a lower rate of silent change, whereas the CRR in partition 1 is very low. When evolutionary rates vary among silent sites, *dn/ds *ratios are commonly interpreted as measures of the *difference *in selection pressure between replacement and silent sites [33] and we propose that the CRR ratio should be interpreted similarly.

**Figure 2:**
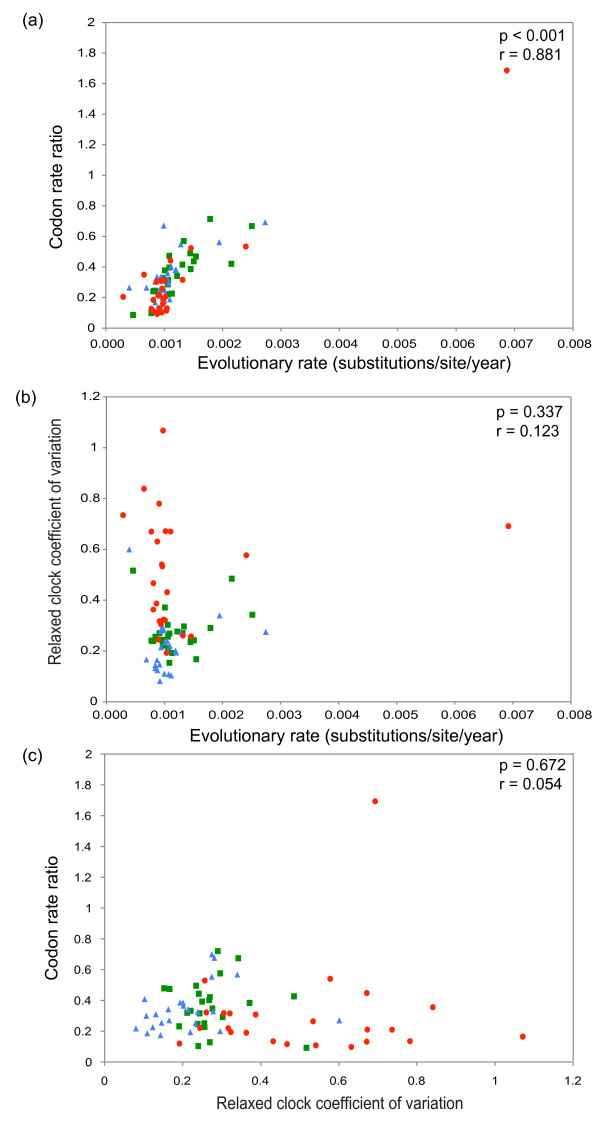
**Scatterplots of the three molecular clock parameter estimates obtained for each partition, calculated using the uncorrelated lognormal (UCLN) relaxed clock model**. Colouring is the same as that in Figure 1: among host subtype 1a data set (green squares), among host subtype 1b data set (blue triangles) and within-host data set (red circles). (a) Plot of mean evolutionary rate versus the coefficient of variation. (b) Plot of mean evolutionary rate versus the codon rate ration. (c) Plot of the coefficient of variation versus the codon rate ration. See main text for full description of each parameter. For each plot, the correlation coefficient (r) and statistical significance (p) of the relationships are given.

Visual inspection of Figure 1 suggests that genomic partitions with high evolutionary rates also have a high CRR. To test for correlations among the three molecular clock parameters (mean rate, COV, and CRR), Figure 2 shows scatterplots among all estimated parameter values. There is a clear positive correlation between mean rate and CRR (Figure 2a; *p *< 0.001) which is robust to the exclusion of the outlying data point (*p *< 0.001). In contrast, there is no clear correlation between mean rate and COV (Figure 2b; *p *= 0.34), nor between CRR and COV (Figure 2c; *p *= 0.67). Figures 2b and 2c do, however, clearly illustrate the higher COV values for the within-host data set.

**Figure 3:**
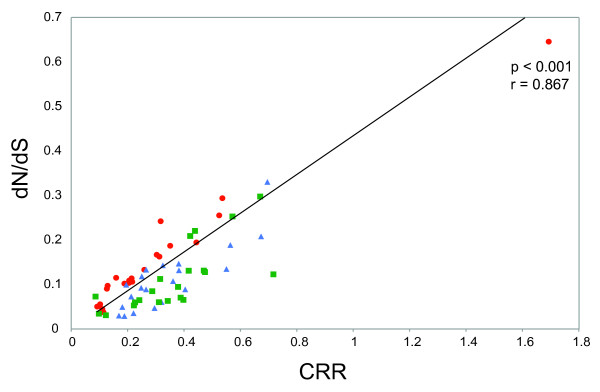
**Scatterplot of the codon rate ratio estimate for each partition, versus the average dn/ds ratio, calculated using PAML**. Colouring is the same as that in Figure 1: among host subtype 1a data set (green squares), among host subtype 1b data set (blue triangles) and within-host data set (red circles).

Lastly, to investigate the relationship between CRR and the more widely-used *dn/ds *ratio, we estimated *dn/ds *values for each partition and data set using PAML [30] (see Methods). Figure 3 shows the scatterplot of *dn/ds *versus CRR values, which, as expected, are strongly correlated (*p *< 0.001). The estimated regression relationship between these two variables is *dn/ds *= 2.44. CRR (assuming no error distribution for the *dn/ds *values; also robust to the exclusion of the outlying data point; *p *< 0.001).

## Discussion

Since its discovery in 1989, the molecular evolution of HCV has been investigated using a wide variety of approaches. Early studies approximated HCV evolutionary rates by simply counting the number of observed changes between sequences sampled at different times from infected patients [34,35] or from a chimpanzee [36]. Subsequent studies used nucleotide substitution models to estimate genetic distances, but commonly focused only on sub-genomic regions, particularly the *Core*, *E1/E2 *and *NS5B *genes (e.g. [14,37]). Rates of evolution have been estimated using (i) relative-rate methods (e.g. Ina *et al *[38]), (ii) linear regressions of genetic distances against sampling times (e.g. Tanaka *et al. *[39]), (iii) maximum likelihood phylogenetic methods (e.g.[15]) and (iv) Bayesian phylogenetic methods (e.g. [40]). Some analyses were performed on among-host data, some on within-host sequences, and others on a combination of both. Unsurprisingly, estimates of the tempo of HCV evolution from these various studies have been highly variable and are impossible to compare directly due to the different methodologies and genome regions employed.

Our study of HCV molecular evolution has employed powerful statistical methods [20] in a consistent manner, enabling us to make reliable inferences about how HCV sequence evolution varies among genome regions, and how it varies between different levels of organisation. As expected, the variation we observed in evolutionary rate across the genome closely matches genomic variation in overall HCV genetic diversity (previously measured in [11,41]). While most of the HCV genome evolves at ~0.001 substitutions/site/year, a rate very typical of RNA viruses [42], the E1/E2 region (partitions 3 and 4) exhibited the highest evolutionary rates, consistent with previous reports (e.g. Itakura *et al. *[23]). This region contains several known antibody epitopes and hyper-variable regions, and our high CRR and *dn/ds *values in these regions confirm the action of positive selection [5,8,23].

A striking result from this study was the elevated evolutionary rate in the intra-host dataset for partition 3 when compared to the among-host dataset. Several explanations are possible. First, we might hypothesise that saturation of nucleotide changes (and hence underestimation of genetic distance) is occurring at the among-host level but not at the within-host level. Although this phenomenon is certainly important for rapidly-evolving viruses when evolution measured over several decades is extrapolated to thousands of years (e.g.[43]), it is very unlikely to be responsible for our result, since our within-host and among-host timescales differ only by a factor of 3. Additionally, we found no evidence of saturation at the 3^rd ^codon position in partitions 3 and 4 for the within-host dataset (data not shown). A second explanation follows from the primary difference between the within- and among-host datasets: the latter contains transmission events while the former does not. Upon transmission to a new host, specific mutations that conferred a fitness advantage in the immune environment of the donor may be lost or quickly revert to wild-type in the new environment of the recipient. This phenomenon has been reported for both HCV [44-47] and HIV [48] and is consistent with the observation of an elevated rate in the epitope-rich region (*E1/E2*) of the HCV genome when transmission bottlenecks are absent (i.e. the within-host dataset). Reversion is also consistent with the elevated CRR in this region, which indicates a higher net rate of adaptation within hosts. A third and intriguing possibility is that slowly-evolving lineages within a host are preferentially-transmitted, resulting in a lower long-term evolutionary rate [1]. This is consistent with the high among-branch rate variation (COV) observed within-hosts here, and for HIV-1 elsewhere [19], suggesting that there is significant variation in the rate of evolution of different lineages within an infected host. However, preferential transmission of slower-evolving lineages should result in a lower among-host long-term replication rate (and thus a lower 3^rd ^codon position rate) equally across all partitions - which is not observed in our data (Additional file 3, Figure S2).

We did observe a consistently higher genome-wide among-host evolutionary rate for subtype 1a in comparison with subtype 1b. This difference could be a consequence of the major modes of transmission of that characterise each subtype: subtype 1a is more commonly associated with intravenous drug use and subtype 1b with past blood transfusions. If within-host HCV evolution is faster at the start of chronic infection (possibly due to adaptation to the new host; [41]) then the long-term among-host rate of evolution will depend to some extent on the rate of transmission [24]. A similar phenomenon, albeit more extreme, has been previously reported for HTLV-II, for which differences in the rate of transmission among different risk groups greatly affect the long-term evolutionary rate [49]. Our data cannot indicate whether average evolutionary rates vary during the course of a single chronic infection, and this question remains an important area for future research.

A higher rate evolution for subtype 1a was especially pronounced for partitions 16 and 17, which contain functionally important genome regions within the NS5a gene. Interestingly, this elevated rate resulted from an increase in 1+2^nd ^substitutions, suggesting the presence of selected sites for this subtype. NS5a forms a necessary part of the replication complex but its function is not fully understood [50]. It is involved in cellular pathways including the interferon response [51] and genetic variation within NS5a has been associated with response to anti-viral drug therapy. Specifically, partition 16 contains the interferon sensitivity-determining region (ISDR), variation within which is reported to predict response to interferon drug therapy [52], although inconsistently between subtypes 1a and 1b [53] and with different results observed in Japanese and European cohorts [50]. The PKR binding domain (which includes the ISDR) and the "V3" region, are also included in partition 16, changes within which may be related to treatment outcome [54]. Similarly, partition 17 contains the less well-studied interferon/ribavirin-resistance determining region (IRRDR) [55], in which excess mutations in patients infected with subtype 1b were more likely to respond to therapy [56]. The possible difference in selective pressure between 1a and 1b that we observed in this region is consistent with subtype-specific differences in resistance mutations in NS5a against the potent viral inhibitor BMS-790052 [57]. Information on drug treatment was unavailable for the subjects included in this study, and although differences in treatments are unlikely to account for the observed differences in this region, the possibility cannot be excluded.

## Conclusions

Because our study employed powerful statistical methods on whole genome sequences, we have been able to quantify the variation in HCV evolutionary dynamics at different scales of organisation for the first time, and thereby confirm that scale-dependent differences in rate are not restricted to HIV and may represent a common feature of chronic RNA viral infection. We posit that the most likely explanation of our current data is that host-specific reversion events are responsible for an elevated rate of evolution and adaptation in the *E1/E2 *region within-hosts compared to among-hosts.

## Authors' contributions

OGP conceived the project. RRG and JP implemented analyses. RRG, JP and OGP interpreted data and wrote the manuscript. PL, AK, and MS provided assistance in running the analyses and interpreting data. All authors read and approved the manuscript.

## Supplementary Material

Additional file 1 **Table S1: Accession numbers and dates of sampling**. Accession numbers and isolate sampling dates of all sequences used in this study. Click here for file

Additional file 2 **Figure S1: The G/C content at each codon position for three datasets**. Three separate data sets are shown: among host subtype 1a (green), among host subtype 1b (blue) and within-host (red). Separate parameters were estimated for each of 21 partitions spanning the HCV coding region (see genome schematic and partition numbering at top of Figure). The alternating white and grey bars are for visual clarity only. Click here for file

Additional file 3 **Figure S2: Absolute rates for the 1^st^+2^nd ^versus 3^rd ^codon positions for each partition**. Three separate data sets are shown: among host subtype 1a (green), among host subtype 1b (blue) and within-host (red). Squares represent the rate of the 1st+2^nd ^positions, circles the 3^rd ^position. The symbols are offset within each partition for visual clarity only. Separate parameters were estimated for each of 21 partitions spanning the HCV coding region (see genome schematic and partition numbering at top of Figure). Click here for file
