# Supplementary material for: The mode and tempo of hepatitis C virus evolution within and among hosts
Source: BMC Evol Biol. 2011 May 19;11:131. doi: 10.1186/1471-2148-11-131 (PMC3112090; doi:10.1186/1471-2148-11-131)
Supplement: Additional file 1 — Table S1: Accession numbers and dates of sampling. Accession numbers and isolate sampling dates of all sequences used in this study. [file 1471-2148-11-131-S1.DOC]

| Dataset | Accession number | Year of Collection |
| --- | --- | --- |
| 1A | EU255987 | 1989 |
| 1A | EU255991 | 1989 |
| 1A | EU255968 | 1989 |
| 1A | EU569722 | 1989 |
| 1A | EU482852 | 1989 |
| 1A | EU234064 | 1990 |
| 1A | EU255989 | 1990 |
| 1A | EU255982 | 1990 |
| 1A | EU255973 | 1990 |
| 1A | EU255984 | 1990 |
| 1A | EU255978 | 1991 |
| 1A | EU255985 | 1991 |
| 1A | EU255977 | 1991 |
| 1A | EU155340 | 1991 |
| 1A | EU255979 | 1991 |
| 1A | EU255964 | 1992 |
| 1A | EU234063 | 1992 |
| 1A | EU155341 | 1992 |
| 1A | EU255970 | 1992 |
| 1A | EU862827 | 1993 |
| 1A | EU255975 | 1994 |
| 1A | EU255988 | 1995 |
| 1A | EU155343 | 1996 |
| 1A | EU155338 | 1996 |
| 1A | EU255966 | 1997 |
| 1A | EU482846 | 2000 |
| 1A | EU155312 | 2000 |
| 1A | EU256053 | 2001 |
| 1A | EU595697 | 2001 |
| 1A | EU482854 | 2001 |
| 1A | EU239713 | 2001 |
| 1A | EU595698 | 2001 |
| 1A | EU255999 | 2002 |
| 1A | EU482871 | 2002 |
| 1A | EU155345 | 2002 |
| 1A | EU256025 | 2002 |
| 1A | EU155323 | 2002 |
| 1A | EU255948 | 2003 |
| 1A | EU155277 | 2003 |
| 1A | EU660385 | 2003 |
| 1A | EU660387 | 2003 |
| 1A | EU255944 | 2003 |
| 1A | EU155314 | 2004 |
| 1A | EU482872 | 2004 |
| 1A | EU256106 | 2004 |
| 1A | EU256107 | 2004 |
| 1A | EU482832 | 2004 |
| 1A | EU256041 | 2005 |
| 1A | EU569723 | 2005 |
| 1A | EU155244 | 2005 |
| 1A | EU155247 | 2005 |
| 1A | EU155354 | 2005 |
| 1A | EU155268 | 2006 |
| 1A | EU482858 | 2006 |
| 1A | EU256046 | 2006 |
| 1A | EU155283 | 2006 |
| 1A | EU482842 | 2006 |
| 1A | FJ390399 | 2007 |
| 1A | EU255935 | 2007 |
| 1A | EU155238 | 2007 |
| 1A | FJ205867 | 2007 |
| 1A | FJ182001 | 2007 |
| 1A | FJ390395 | 2008 |
| 1A | FJ410172 | 2008 |
| 1A | FJ390394 | 2008 |
| 1B | EU155333 | 1989 |
| 1B | EU482849 | 1989 |
| 1B | EU155334 | 1990 |
| 1B | EU155337 | 1990 |
| 1B | EU155331 | 1990 |
| 1B | EU255961 | 1991 |
| 1B | EU155325 | 1991 |
| 1B | EU234061 | 1991 |
| 1B | EU255962 | 1991 |
| 1B | EU234062 | 1992 |
| 1B | EU155328 | 1992 |
| 1B | EU155324 | 1992 |
| 1B | EU482888 | 1992 |
| 1B | EU155327 | 1992 |
| 1B | EU155330 | 1994 |
| 1B | EU155332 | 1996 |
| 1B | FJ478453 | 1999 |
| 1B | EU155280 | 2001 |
| 1B | EU239714 | 2001 |
| 1B | EU256054 | 2001 |
| 1B | EU155302 | 2001 |
| 1B | EU482877 | 2001 |
| 1B | EU155219 | 2002 |
| 1B | EU529682 | 2002 |
| 1B | EU155367 | 2002 |
| 1B | EU155366 | 2002 |
| 1B | EU256081 | 2002 |
| 1B | EU256045 | 2003 |
| 1B | EU155308 | 2003 |
| 1B | EU155304 | 2003 |
| 1B | EU256066 | 2003 |
| 1B | EU155356 | 2003 |
| 1B | EU155229 | 2004 |
| 1B | EU155372 | 2004 |
| 1B | EU155358 | 2004 |
| 1B | EU155217 | 2004 |
| 1B | EU256076 | 2004 |
| 1B | EU155370 | 2005 |
| 1B | EU155371 | 2005 |
| 1B | EU256078 | 2005 |
| 1B | EU256059 | 2005 |
| 1B | EU482874 | 2005 |
| 1B | EU482885 | 2006 |
| 1B | EU155253 | 2006 |
| 1B | EU155365 | 2006 |
| 1B | EU155264 | 2006 |
| 1B | EU256091 | 2006 |
| 1B | EU155234 | 2006 |
| 1B | FJ024277 | 2007 |
| 1B | FJ024279 | 2007 |
| 1B | FJ390396 | 2007 |
| 1B | FJ024086 | 2007 |
| 1B | FJ390397 | 2008 |
| 1B | FJ390398 | 2008 |
| Anti-D | AF313916 | 1977.25 |
| Anti-D | AB154177 | 1994.33 |
| Anti-D | AB154178 | 1994.4 |
| Anti-D | AB154179 | 1994.42 |
| Anti-D | AB154180 | 1994.42 |
| Anti-D | AB154181 | 1994.47 |
| Anti-D | AB154182 | 1995.25 |
| Anti-D | AB154183 | 1996.31 |
| Anti-D | AB154184 | 1996.52 |
| Anti-D | AB154185 | 1996.53 |
| Anti-D | AB154186 | 1996.53 |
| Anti-D | AB154187 | 1996.59 |
| Anti-D | AB154188 | 1996.72 |
| Anti-D | AB154189 | 1997.13 |
| Anti-D | AB154190 | 1997.29 |
| Anti-D | AB154191 | 1997.43 |
| Anti-D | AB154192 | 1997.47 |
| Anti-D | AB154193 | 1998.2 |
| Anti-D | AB154194 | 1999.11 |
| Anti-D | AB154195 | 1999.13 |
| Anti-D | AB154196 | 1999.25 |
| Anti-D | AB154197 | 1999.25 |
| Anti-D | AB154198 | 1999.3 |
| Anti-D | AB154199 | 1999.37 |
| Anti-D | AB154200 | 1999.86 |
| Anti-D | AB154201 | 1999.9 |
| Anti-D | AB154202 | 1999.94 |
| Anti-D | AB154203 | 2000.09 |
| Anti-D | AB154204 | 2000.26 |
| Anti-D | AB154205 | 2000.36 |
| Anti-D | AB154206 | 2000.39 |
