# Supplementary figures and images for: The mode and tempo of hepatitis C virus evolution within and among hosts
Source: BMC Evol Biol. 2011 May 19;11:131. doi: 10.1186/1471-2148-11-131 (PMC3112090; doi:10.1186/1471-2148-11-131)

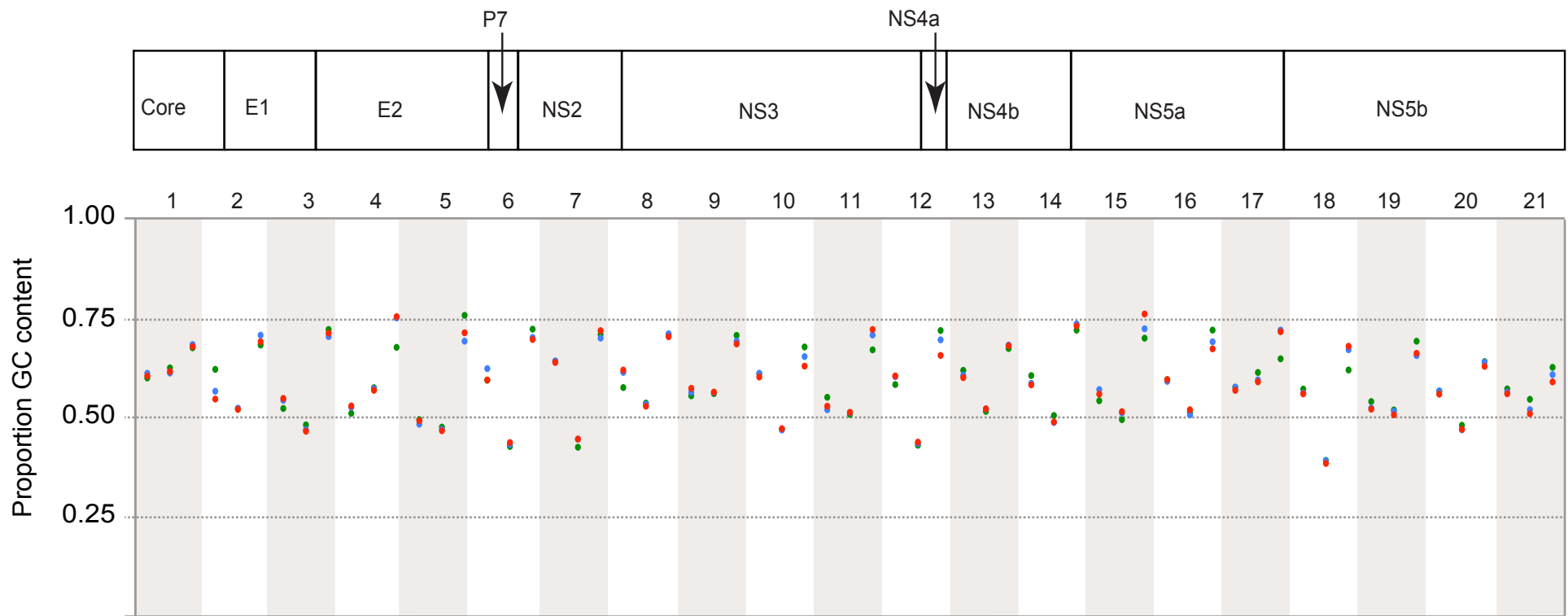

Supplement: Additional file 2 — Figure S1: The G/C content at each codon position for three datasets. Three separate data sets are shown: among host subtype 1a (green), among host subtype 1b (blue) and within-host (red). Separate parameters were estimated for each of 21 partitions spanning the HCV coding region (see genome schematic and partition numbering at top of Figure). The alternating white and grey bars are for visual clarity only. [file 1471-2148-11-131-S2.PDF]

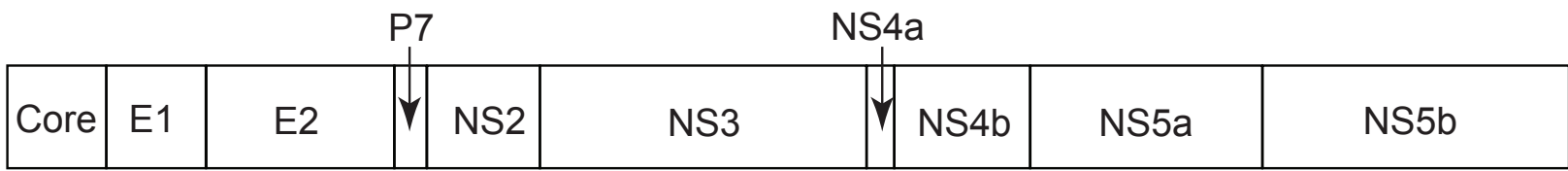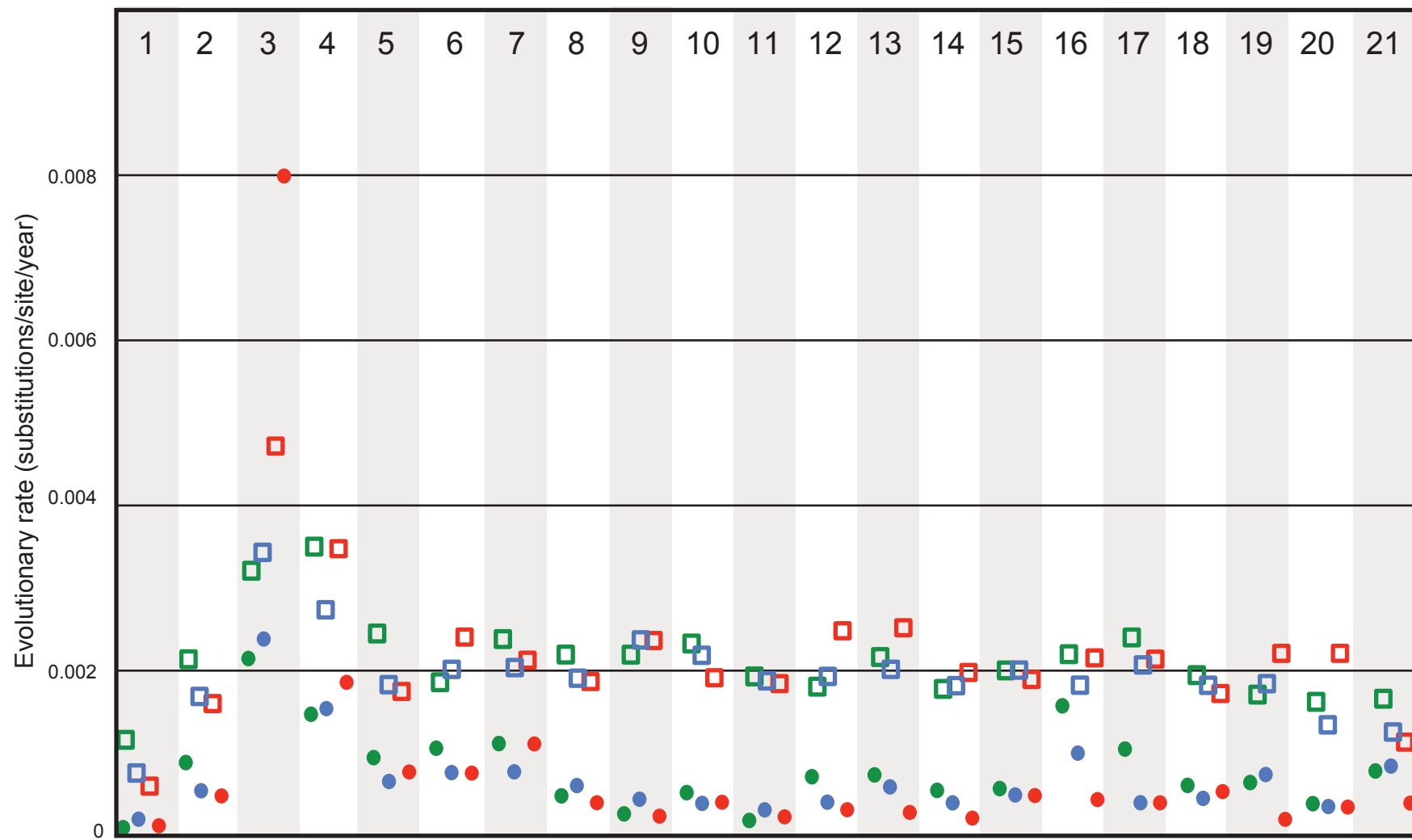

Supplement: Additional file 3 — Figure S2: Absolute rates for the 1st+2nd versus 3rd codon positions for each partition. Three separate data sets are shown: among host subtype 1a (green), among host subtype 1b (blue) and within-host (red). Squares represent the rate of the 1st+2nd positions, circles the 3rd position. The symbols are offset within each partition for visual clarity only. Separate parameters were estimated for each of 21 partitions spanning the HCV coding region (see genome schematic and partition numbering at top of Figure). [file 1471-2148-11-131-S3.PDF]
